# One-Carbon Metabolism and Alzheimer’s Disease: Focus on Epigenetics

**DOI:** 10.2174/138920210791233090

**Published:** 2010-06

**Authors:** Fabio Coppedè

**Affiliations:** Department of Neuroscience, University of Pisa, Via Roma 67, 56126 Pisa, Italy

**Keywords:** Alzheimer’s disease, Epigenetics, folate metabolism, homocysteine, folate gene polymorphisms, SAM, SAH, MTHFR.

## Abstract

Alzheimer’s disease (AD) represents the most common form of dementia in the elderly, characterized by progressive loss of memory and cognitive capacity severe enough to interfere with daily functioning and the quality of life. Rare, fully penetrant mutations in three genes (*APP*, *PSEN1* and *PSEN2*) are responsible for familial forms of the disease. However, more than 90% of AD is sporadic, likely resulting from complex interactions between genetic and environmental factors. Increasing evidence supports a role for epigenetic modifications in AD pathogenesis. Folate metabolism, also known as one-carbon metabolism, is required for the production of S-adenosylmethionine (SAM), which is the major DNA methylating agent. AD individuals are characterized by decreased plasma folate values, as well as increased plasma homocysteine (Hcy) levels, and there is indication of impaired SAM levels in AD brains. Polymorphisms of genes participating in one-carbon metabolism have been associated with AD risk and/or with increased Hcy levels in AD individuals. Studies in rodents suggest that early life exposure to neurotoxicants or dietary restriction of folate and other B vitamins result in epigenetic modifications of AD related genes in the animal brains. Similarly, studies performed on human neuronal cell cultures revealed that folate and other B vitamins deprivation from the media resulted in epigenetic modification of the *PSEN1* gene. There is also evidence of epigenetic modifications in the DNA extracted from blood and brains of AD subjects. Here I review one-carbon metabolism in AD, with emphasis on possible epigenetic consequences.

## INTRODUCTION

### 1. Alzheimer's Disease

Alzheimer's disease (AD) is a complex multi-factorial neurodegenerative disorder and represents the most common form of dementia in the elderly. In 2006, the worldwide prevalence of AD was 26.6 million. It has been estimated that following the global aging of the world’s population this number will quadruple by 2050, suggesting that 1 in 85 persons worldwide will be living with the disease [1]. AD is the sixth leading cause of all deaths in the United States, and the fifth leading cause of death in Americans aged 65 years and older. It is estimated that 5.3 million Americans have AD, and that every 70 seconds someone in America develops AD; by 2050, this time is expected to decrease to every 33 seconds [2]. No striking racial differences appear in AD prevalence or incidence and no geographic isolates of the disease are known [3].

AD is clinically characterized by a progressive neurodegeneration in selected brain regions, including the temporal and parietal lobes and restricted regions within the frontal cortex and the cingulate gyrus, resulting in gross atrophy of the affected regions and leading to memory loss accompanied by changes of behaviour and personality severe enough to affect work, lifelong hobbies or social life. Affected brain regions are also characterized by the occurrence of extracellular amyloid deposits or senile plaques (SP) and by the presence of neurofibrillary tangles (NFT) composed of intraneuronal aggregates of hyperphosphorylated tau protein [4]. The disease gets worse over time, and it is fatal. Unfortunately, currently used treatments offer a small symptomatic benefit, but no treatments to delay or halt the progression of the disease are as yet available [5].

One of the most important early discoveries in understanding the etiology of AD was that the primary component of the extracellular amyloid deposits in AD brains is an approximately 40-residue long peptide, known as amyloid β (Aβ) peptide. It was subsequently established that Aβ is the product of the proteolytic processing of its precursor, the amyloid precursor protein (APP). APP can be processed by α-secretase and γ-secretase (a protein complex composed by presenilins and other proteins) producing non-amyloidogenic peptides, or by β-secretase (β-site APP cleaving enzyme 1, BACE1) and (γ-secretase producing Aβ peptides. Therefore the balance between different secretase activities is very important in the maintenance of the physiological levels of non-amyloidogenic and amyloidogenic fragments. The two major forms of Aβ that are produced by APP processing under normal conditions are 40 and 42 residues in length (Aβ_40_ and Aβ_42_, respectively). Aβ_42 _is the major component of SP. In a normal individual the majority of Aβ produced is of the shorter variety, Aβ_40_; whereas mutations causing familial AD lead to increased Aβ_42_ production or increase the Aβ_42_/Aβ_40_ ratio without increasing Aβ_42_ production [6,7]. 

Rare mutations in *APP*, presenilin-1 (*PSEN1*) and presenilin-2 (*PSEN2*) genes cause early-onset (< 65 years) familial forms of the disease accounting for less than 1% of the total AD cases [8]. As described above, AD causative mutations lead to altered APP production and/or processing and the disease is transmitted in families following a Mendelian inheritance pattern [6,7]. Importantly, 50% or more of early-onset AD cases are not explained by the known *APP, PSEN1 *and *PSEN2* mutations, suggesting the existence of yet unknown genetic factors [8]. Furthermore, the existence of late-onset AD families (> 65 years) with an apparent autosomal dominant pattern of transmission suggests the presence of other Mendelian mutations with less aggressive phenotypes [8]. However, the vast majority of AD cases (90-95%) are sporadic; they are diagnosed in people over 65 years of age, and are referred as late-onset sporadic forms, likely resulting from the interaction between genetic, epigenetic, environmental and stochastic factors [9]. Several hundreds of genes have been investigated in genetic association studies as possible AD susceptibility or modifier genes, and more-recent genome-wide association studies are revealing novel polymorphisms that could account for increased AD risk; however, only the apolipoprotein E (APOE) ε4 allele is a validated AD risk factor [9]. In parallel several environmental agents, including metals, pesticides, dietary factors and brain injuries, have been suggested as possible AD environmental risk factors [9,10]. However, despite active research in the field the etiology of sporadic AD cases is still uncertain. 

Folate metabolism, also known as one-carbon metabolism, is required for the production of S-adenosylmethionine (SAM), which is the major DNA methylating agent [11,12]. AD individuals are characterized by decreased plasma folate values, as well as increased plasma homocysteine (Hcy) levels, and there is indication of impaired SAM levels in AD brains [13,14]. In this review article I discuss one-carbon metabolism in AD individuals, with emphasis on possible epigenetic modifications of the promoters of AD-related genes.

### 2. One-Carbon Metabolism: An Overview

Folates are essential nutrients required for one-carbon biosynthetic and epigenetic processes. They are derived entirely from dietary sources, mainly from the consumption of green vegetables, fruits, cereals, and meat. Folic acid is the synthetic form added to foods and found in dietary supplements. After intestinal absorption, folate metabolism requires reduction and methylation into the liver to form 5-methyltetrahydrofolate (5-MTHF), release into the blood and cellular uptake; then it can be used for the synthesis of DNA and RNA precursors or for the conversion of homocysteine (Hcy) to methionine, which is then used to form SAM. Folic acid is converted to a natural biological form of the vitamin as it passes through the intestinal wall, with enzymatic reduction and methylation resulting in the circulating form of the vitamin, 5-MTHF [12].

Folate do not cross biological membranes by diffusion alone, but requires several transport systems to enter the cells, the best characterized being the reduced folate carrier (RFC1). Methylenetetrahydrofolate reductase (MTHFR) is the first enzyme in the DNA methylation pathway since it reduces 5,10-methylentetrahydrofolate (5,10-MTHF) to 5-MTHF. Subsequently, methionine synthase (MTR) transfers a methyl group from 5-MTHF to Hcy forming methionine and tetrahydrofolate (THF). Methionine is then converted to SAM in a reaction catalyzed by methionine adenosyltransferase (MAT). Most of the SAM generated is used in transmethylation reactions, whereby SAM is converted to S-adenosylhomocysteine (SAH) by transferring the methyl group to diverse biological acceptors, including proteins and DNA. Vitamin B12 (or cobalamin) is a cofactor of MTR, and methionine synthase reductase (MTRR) is required for the maintenance of MTR in its active state. If not converted into methionine, Hcy can be condensed with serine to form cystathionine in a reaction catalyzed by cystathionine (β-synthase (CBS), which requires vitamin B6 as a cofactor. Cystathionine can be then utilized to form the antioxidant compound glutathione (GSH). Another important function of folates is in the de novo synthesis of DNA and RNA precursors, required during nucleic acid synthesis and for DNA repair processes. Therefore, depending on cellular demands 5,10-MTHF can be used for the synthesis of SAM or for the synthesis of nucleic acid precursors, and the folate metabolic pathway is tightly regulated by intracellular levels of metabolites and cofactors [11,12]. A diagram illustrating folate metabolism is shown in Fig. (**1**).

## ONE-CARBON METABOLISM IN ALZHEIMER’S DISEASE

### 1. Homocysteine, Folate and other B Vitamins

Several investigators have measured plasma values of folate, Hcy, vitamin B12 and vitamin B6 in AD subjects and healthy matched controls [15-36]. Most of these studies are shown (Table **1**). Overall, the majority of the studies agree that plasma Hcy values are increased in AD subjects [15,16,18,19,21-24,26-34,36]; there is also indication that folate values are reduced in the plasma of AD individuals respect to controls, and the difference reached significance in several studies [16,19,21-23,26,33-36]. Less data have been obtained on vitamins B6 and B12, and results are still inconclusive [15,16,18,19,21-23,25-27,29,33]. However, some authors observed significantly decreased levels of vitamin B12 in plasma of AD subjects respect to controls [19,22,26,33]. There is also some indication that Hcy levels are increased in the cerebrospinal fluid (CSF) of AD patients, respect to controls [22,37,38]. Particularly, Selley and co-workers measured the concentrations of Hcy, vitamin B12 and folate in the CSF of 8 patients with AD and 6 control subjects. The concentrations of Hcy resulted significantly higher in the CSF of AD patients than in controls. There was also a significant positive correlation between the plasma concentration of Hcy and the CSF concentrations of Hcy [22]. Similarly, Hasegawa and co-workers observed significantly increased CSF concentrations of Hcy in AD patients respect to controls [37]. Isobe and co-workers measured total Hcy levels in the CSF of 17 AD patients, 16 individuals with Parkinson’s disease (PD), and 16 control subjects, observing that respect to controls both AD and PD subjects had an average of 31% increased Hcy levels [38]. However, others measured CSF total Hcy levels in 22 normal elderly subjects and 38 AD patients, observing no difference between the two groups [39].

### 2. S-Adenosylmethionine, S-Adenosylhomocysteine and Methionine Adenosyltransferase

Several studies have been performed to measure SAM and SAH levels, as well as MAT activity, in plasma, CSF and brain regions of AD subjects (Table **2**). In 1990 Bottiglieri and co-workers observed a significant 41% reduction in SAM levels in the CSF of 9 AD subjects respect to the levels observed in 13 control individuals. Moreover, oral SAM treatment (1200 mgs daily) for 4 to 8 months was associated with a significant increase in CSF SAM in AD patients [40]. Subsequently, Morrison and co-workers measured SAM and SAH levels in autopsied brains of 11 AD subjects and 14 controls. All the experiments were performed over a 15-hour post-mortem interval in tissues obtained from frontal cortex, occipital cortex, temporal cortex and hippocampus. As compared with the controls, mean SAM and SAH levels were significantly reduced in all the areas of AD brains examined (from -56 to -85%). The authors also measured the activity of methionine adenosyltransferase in a subgroup of 5 AD brains and 5 control brains, observing normal MAT activity in AD temporal and occipital cortices [14]. By contrast, others observed a decreased MAT activity in erytrocytes of 9 AD patients respect to 10 controls; the decreased MAT activity in AD patients also correlated with increased serum Hcy levels. Treatment of AD subjects for 6 months with vitamin B12 (1mg x 7days + 1mg/week), SAM (200 mg twice daily) and folate (2.5 mg every two days) caused a significant decrease in Hcy levels that was paralleled by a significant increase in MAT activity [41]. Alterations of MAT activity have been also found in erytrocytes of vitamin B12-deficient AD patients, and in the brain of AD subjects [42]. Subsequent studies in SH-SY5Y neuroblastoma cells have demonstrated that the MAT catalytic activity was inversely correlated to methionine concentrations [43]. A subsequent study performed on 30 AD patients and 28 controls failed to find statistical differences in SAM, SAH and 5-MTHF levels and in SAM/SAH ratio in the CSF of AD patients and age-matched controls [44]. On the contrary, a significant increase in the plasma concentrations of SAH, Hcy and SAM was observed in AD patients [45]. SAH binds to the catalytic region of methyltransferases with higher affinity than SAM and is a potent inhibitor of cellular methylation. SAH is hydrolyzed to Hcy and adenosine by the enzyme SAH hydrolase [46]. Hcy is an inhibitor of SAH hydrolase and increased Hcy concentrations result in parallel increases in intracellular SAH and inhibition of methyltransferases [47-49]. It was observed that increased Hcy concentrations are associated with decreased concentrations of adenosine in the plasma of AD individuals, likely due to the inhibition of SAH hydrolase and increased production of SAH [50]. It has been also reported that increased SAH concentrations in the brains of AD patients inhibit methyltransferases and that this was related to cognitive impairment [51]. 

### 3. Polymorphisms in Folate/Homocysteine Metabolizing Genes and Risk of Alzheimer’s Disease

**Fig. (1). Overview of the folate metabolic pathway:**
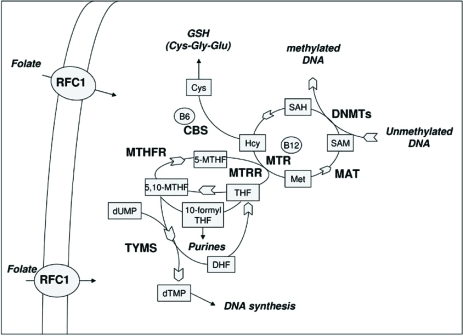
*Metabolites:* Cys = cysteine; dTMP = deoxythymidine monophosphate; dUMP = deoxyuridine monophosphate; DHF = dihydrofolate; 10- formyl-THF = 10- formyl-tetrahydrofolate; GSH = glutathione; Hcy = homocysteine; Met = methionine; 5-MTHF = 5- methyltetrahydrofolate; 5,10-MTHF = 5,10-methylentetrahydrofolate; SAH = S-adenosylhomocysteine; SAM = S-adenosylmethionine; THF = tetrahydrofolate *Enzymes:* CBS = cystathionine β-synthase; DNMTs = DNA methyltransferases; MAT = methionine adenosyltransferase; MTHFR = methylenetetrahydrofolate reductase; MTR = methionine synthase; MTRR = methionine synthase reductase; RFC1 = reduced folate carrier. *Cofactors:* B6 = vitamin B6; B12 = vitamin B12.

Polymorphisms of genes participating in one-carbon metabolism have been largely investigated as candidate AD risk factors (Table **3**). Methylenetetrahydrofolate reductase is the flavoprotein that catalyzes the conversion of 5,10-methylentetrahydrofolate (5,10-MTHF) to 5-methylTHF (Fig. **1**). The *MTHFR *gene has been largely studied in AD association studies. Particularly, two common *MTHFR* polymorphisms, namely 677C>T (Ala222Val) and 1298A>C (Glu429Ala), are known to reduce MTHFR activity [52]. Numerous studies have shown that the *MTHFR* 677T allele is associated with increased total plasma Hcy levels (tHcy) and decreased serum folate levels, mainly in 677TT homozygous subjects [53-55]. Several authors investigated the *MTHFR *677C>T polymorphism as a candidate AD risk factor, but results are still conflicting including either positive and negative associations [19,30,31,36,56-65]. Some authors observed that *MTHFR* 677TT homozygous AD subjects had higher plasma tHcy values and/or decreased folate values compared to carriers of the *MTHFR *677CT or 677CC genotypes [19,30,59,61,64]. Others observed interaction between the *MTHFR *677T allele and the *APOE *genotype in modifying AD risk [36,61,62,65]. The *MTHFR *1298A>C polymorphism has been studied less extensively than the 677C>T in AD association studies, and results are still conflicting [31,58,61,66,67]. *MTHFR* 677C>T and 1298A>C polymorphisms are in strong linkage disequilibrium (LD), particularly the 677T allele has been nearly always observed in *cis* with the 1298C allele. A study suggested that the 677T variant arose later than the 1298C variant on a chromosome harbouring 1298A [68]. LD is not complete; however frequencies below 0.005 have generally been reported for the rare 677T–1298C haplotype [69]. A biological explanation for the LD existing between the two different *MTHFR *polymorphisms has been recently suggested [70]. MTHFR works as a dimer and monomers associate head to tail, but the stability of the dimer depends on what aminoacid is present at position 222 and what at position 429, resulting from the *MTHFR* 677/1298 genotype. Based on this model it was proposed that the combined presence of both polymorphisms in homozygosis would impair significantly the stability and the activity of the dimer protein [70]. Wakutani and co-workers [71] investigated *MTHFR *haplotypes generated by the combinations of three polymorphisms, 677C>T (Ala222Val), 1298A>C (Glu429Ala), and 1793A>G (Arg594Gln), in AD subjects and controls, suggesting that the haplotype 677C/1298C/1793G could be protective against the development of AD [71]. Polymorphisms in the regulatory region of the *MTHFR *gene (-713G>A and -393C>A, upstream of the start codon) were not associated with AD risk [72].

The first report of a *RFC1* gene polymorphism was in 2000 by Chango and co-workers [73] who described a high frequency 80G>A single nucleotide polymorphism resulting in replacement of an arginine by histidine (Arg27His). Authors found a moderate, but significant, increase in tHcy levels in doubly homozygous *RFC1* 80GG/ *MTHFR *677TT subjects as compared to *RFC1* 80GG/ *MTHFR* 677CC or CT subjects. In addition, individuals who were *RFC1 *80AA/*MTHFR* 677CT had higher plasma folate levels than those who were *RFC1 *80GG/ *MTHFR* 677CT [73]. Further studies provided conflicting results, therefore the effect of the *RFC1 *80G>A polymorphism on plasma folate and Hcy levels is still debated [74-77]. Bi and co-workers have recently investigated *RFC1 *80G>A and *MTHFR *677C>T polymorphisms in a large cohort of AD patients and controls. Significant associations of the *RFC1* 80G allele and GG genotype with AD risk was found. However, no interaction between the two studied polymorphisms was found, nor the *RFC1* 80G variant was associated with plasma folate and Hcy levels [36]. Women who had a Down syndrome (DS) child at a young age have a five-fold increased risk to develop AD later in life, respect to control women [78,79]. We recently observed that the *RFC1* 80GG/ *MTHFR *677TT genotype is more frequent in young mothers of DS children than in control women, while the *RFC1 *80 (AA or GA)/ *MTHFR *1298AA genotype is more frequent in control mothers [80]. Moreover, we observed that young mothers of DS individuals have an increased frequency of micronuclei (mainly originating from chromosome malsegregation events, including malsegregation of chromosome 21) in peripheral blood cells respect to control mothers and that *MTHFR *677TT subjects had the highest levels of chromosome damage [81-83]. Similarly, an increased frequency of micronuclei and/or a preferential occurrence of chromosome 21 malsegregation has been observed in blood cells, buccal mucosa cells, fibroblasts and neurons of AD patients [84-87]. Several *in vitro* studies have shown that folate depletion and increased Hcy concentrations induce an increased frequency of micronuclei  [89-90], and a recent study performed on 164 healthy individuals of different age showed the lowest percentage of micronuclei in blood cells of *RFC1 *80GG individuals [90].

A common *MTR *2756A>G (Asp919Gly) polymorphism is known, and there is indication from large scale population studies that it can have an effect on Hcy levels [92]. However, results are still conflicting and the contribution of the *MTR *2756A>G polymorphism to Hcy concentrations has not been fully clarified. Some studies reported increased Hcy levels in the presence of the wild type (*MTR* 2756A) allele [93-94], whereas others observed increased Hcy levels in the presence of the mutant (*MTR* 2756G) allele [95,96]. There is also indication that the heterozygous genotype *MTR *2756AG is associated with increased Hcy concentrations in DS individuals [97]. These apparent discrepancies might be explained by recent evidence suggesting that the interaction between different polymorphisms may totally modify their individual effect, and that the same genotype combinations could have different effects on maternal Hcy levels in different individuals, depending on interactions with nutritional and lyfestile factors [98]. In 2003 Beyer and co-workers observed association between the *MTR *2756AA genotype and increased AD risk [99]. Subsequently, Bosco and co-workers observed association of the *MTR *2756AA genotype with dementia severity of sporadic AD [100]. More recently Zhao and co-workers did not reveal significant association between the *MTR* 2756A>G polymorphism and AD. However authors observed a trend between the *MTR *A allele and increased AD risk (*P=*0.09), therefore a weak effect of the A allele on developing AD could not be completely excluded [101].

Vitamin B12, in the form of methylcobalamin, serves as a coenzyme for MTR during the remethylation of Hcy to methionine (Fig. **1**). In circulation, vitamin B12 is bound to two plasma proteins: transcobalamin or haptocorrin. Transcobalamin (TC) is the transport protein required for cellular uptake of vitamin B12. Specific membrane receptors recognize the trancobalamin-vitamin B12 complex, whereas free vitamin B12 or haptocorrin-bound vitamin B12 is not taken up by the cell [102,103]. Several studies have related holo-transcobalamin (holo-TC) levels to AD risk [104-106]. A common* TC* 776C>G polymorphism results in the replacement of proline with arginine (Pro259Arg) and negatively affects vitamin B12 metabolism, thus increasing plasma Hcy levels [107]. Conflicting results have been obtained when investigating the *TC *776C>G polymorphism as a candidate AD risk factor. Zetterberg and co-workers reported that this polymorphism influences holo-TC concentration in the CSF from AD patients [108], and suggested that it could be a modifiable AD genetic risk factor [109]. McCaddon and co-workers observed that serum holo-TC levels were significantly higher in *TC* 776CC individuals and that proportionately fewer *TC* 776CC homozygotes appear to develop AD at any given age [110]. Others failed to find association between the *TC *677C>G polymorphism and sporadic AD risk [61,100].

Human cystathionine (-synthase (CBS) is a hemoprotein which catalyzes the condensation of Hcy and serine to form cystathionine, which is then used to form GSH (Fig. **1**). Insufficiency in CBS activity may lead to hyperhomocysteinemia and a gross deficiency in CBS activity is associated with homocystinuria, an inborn recessive metabolic disorder [111,112]. The *CBS* gene is known to have a large number of mutations, including missense and nonsense ones, as well as some insertion, deletion and splice site variants, some of which are polymorphic in nature [111]. The identification of an 844ins68 insertion in the *CBS* gene was first reported in a patient affected by homocysteinuria due to CBS deficiency [113]. Subsequent studies have revealed that this insertion is not a disease causing mutation but rather a common polymorphism whose frequency is largely different among human populations, with the variant allele being prevalent in African, European and North American populations [114-116]. Several studies report that the *CBS* 844ins68 polymorphism alone has not a relevant effect on tHcy concentrations [117,118]. Beyer and co-workers genotyped 206 AD patients and 186 age-matched controls, observing that the 844ins68 mutation was associated with AD risk in subjects aged 75 years or more at onset [119]. By contrast, Zhang and co-workers observed no difference in the distribution of the *CBS* 844ins68 allele between 105 AD patients and 102 matched controls [30]. Moreover, no association between the polymorphism and plasma Hcy levels was observed [30]. Therefore, the contribution of this polymorphism to AD risk is still controversial.

### 4. Linking One-Carbon Metabolism to Epigenetics

Prospective cohort studies showed that there is substantial evidence to suggest that increased serum Hcy levels predispose to AD [120-122]. There is also indication from prospective cohort studies suggesting that higher folate intake is related to lower AD risk in the elderly [122,123]. On the contrary, significant associations between increased risk of AD and blood levels of vitamin B12 and vitamin B6 were not found [122,124]. Several hypotheses have been formulated to explain the increased AD risk associated with high serum Hcy levels and low serum folate. For istance, folate deficiency fosters a decline in SAM, decreasing DNA methylation during aging and AD [14,120]. Folate deficiency and resultant SAM depletion lead to increased levels of Hcy, which in turn potentiate Aβ peptide toxicity [125]. Hcy is a critical branch point metabolite that can influence cellular levels of SAM and SAH, which regulate the activity of methyltransferases during DNA methylation and posttranslational modification of proteins [126]. Studies in rodents showed that Hcy accumulation reduces cellular levels of SAM, stimulates glutamate excitotoxicity and increases oxidative damage [127]. Hcy has been also associated to vascular disease in AD, with attention focused on vascular changes related to AD as a consequence of Aβ peptide toxicity and its deposition [128]. Several studies suggest a correlation between plasma Hcy concentrations and plasma Aβ levels [129,130]. Moreover, there is indication that elevated Hcy causes tau hyperphosphorilation, NFT formation and SP formation *via *inhibition of methyltransferases and reduced methylation of protein phosphatase 2A [131,132]. However, one of the most exciting hypothesis linking one-carbon metabolism to AD risk suggests that impaired folate/Hcy metabolism and subsequent reduction of SAM levels might result in epigenetic modifications of the promoters of AD-related genes leading to increased Aβ peptide production [133,134]. One of the most studied epigenetic modifications is the change of methylation patterns of CpG rich regions in the promoters of specific genes, resulting in gene silencing (hypermethylation) or overexpression (hypomethylation). In the next section I will discuss evidence from cell cultures, animal models and humans, linking one-carbon metabolism to epigenetic modifications of AD-related genes (Table **4**).

## EPIGENETIC MODIFICATIONS OF AD-RELATED GENES

### 1. Cell Cultures

Several studies performed on neuroblastoma cells suggest that the manipulation of environmental factors can epigenetically modify the expression of AD-related genes and proteins. Particularly, the levels of methylation of CpG islands in the promoters of the *APP* and the *PSEN*1 (Presenilin 1, the core of the (γ-secretase activity that cleaves APP) genes were analyzed on human neuroblastoma SK-N-SH or SK-N-BE cell lines, and it was observed that under conditions of folate and vitamin B12 deprivation from the media, the status of methylation of the promoter of the *PSEN1* gene underwent a variation, with a subsequent deregulation of the production of presenilin1, BACE1 and APP proteins [134]. Both (γ-secretase and β-secretase are required during the amyloidogenic cleavage of APP leading to the formation of Aβ peptides. Therefore, this study confirmed that some of the genes responsible for the production of Aβ peptides in AD can be regulated through an epigenetic mechanism depending on the cellular availability of folate and B12 vitamins, and involving the production of SAM and the status of methylation of CpG islands in the DNA [134]. Moreover, SAM administration in human neuroblastoma SK-N-SH cell cultures resulted in downregulation of *PSEN1* gene expression and Aβ peptide production [133]. To investigate whether SAM administration globally influenced gene expression in the brain, Cavallaro and co-workers analysed 588 genes of the central nervous system in SK-N-BE neuroblastoma cells, observing that only 7 genes were modulated by SAM treatment (and therefore by DNA methylation); 3 were up-regulated and 4 down-regulated [135]. The effects of B vitamin deprivation (folate, vitamin B12 and vitamin B6 deprivation) and SAM addition have been tested using human SK-N-BE neuroblastoma and A172 glioblastoma cell lines. The results indicated that Hcy accumulation induced through vitamin B deprivation could impair the "methylation potential" with consequent presenilin 1, BACE1 and Aβ upregulation. However, Hcy alterations had an effect on neuroblastoma but not on glioblastoma cells [136]. Lin and co-workers examined the hypothesis that SAH may increase the formation of the Aβ peptide in BV-2 mouse microglial cells through hypomethylation of the promoters of genes encoding presenilin 1, APP and BACE1. The results showed that SAH increases the production of A( in BV-2 cells possibly by increased expression of APP and induction of hypomethylation of *APP* and *PSEN1* gene promoters [137]. Recent studies on murine cerebral endothelial cells have demonstrated that Aβ reduces global DNA methylation whilst increasing DNA methylation of the gene encoding neprilysin (NEP), one of the enzymes responsible for Aβ degradation, thus suppressing the NEP expression in mRNA and protein levels [138]. These results indicate that Aβ induces epigenetic effects, suggesting that DNA methylation might be part of a vicious cycle involving the reduction in NEP expression along with a resultant increase in Aβ accumulation, which in turn induces global DNA hypomethylation [138]. 

### 2. Animal Models

A combination of dietary folate, vitamin B12 and vitamin B6 deprivation (B vitamin deprivation) resulted in hyperhomocysteinemia, increased brain SAH levels, depletion of brain SAM, and enhancement of presenilin 1 and BACE1 expression and Aβ deposition in mice [139]. Moreover, B vitamin deprivation induced hypomethylation of specific CpG moieties in the 5'-flanking region of *PSEN1* in mice, and the *PSEN1* promoter methylation status was correlated with gene expression [140]. Dietary deficiency in folate and vitamin E, in condition of oxidative stress (the diet contained iron as a pro-oxidant), increased presenilin 1 expression, (γ-secretase activity, and Aβ levels in normal adult mice. These increases were particularly evident in mice lacking murine apolipoprotein E. Dietary supplementation with SAM in the absence of folate attenuated these deleterious consequences [141] A similar experiment was performed in mice expressing the human *APOE *gene. Mice expressing human apolipoprotein ε4 (associated with increased risk of AD), apolipoprotein ε3, and apolipoprotein ε2 (associated with reduced risk of AD) were subjected to a diet lacking folate and vitamin E, and containing iron as a pro-oxidant. The study revealed that presenilin 1 and (γ-secretase were over-expressed in ε3 mice to the same extent as in ε4 mice, and were not alleviated by SAM supplementation. Aβ increased only in ε4 mice and was alleviated by SAM supplementation [142]. Moreover, the deficient diet increased phosphorylated tau levels (the component of neurofibrillary tangles) in ε4 but not in ε3 mice, which was prevented by SAM supplementation [143]. 

Basha and co-workers exposed rodents to lead (Pb) and monitored the lifetime expression of the APP gene. Authors observed that APP mRNA expression was transiently induced in neonates, but exhibited a delayed over-expression 20 months after exposure to Pb had ceased. This up-regulation in APP mRNA expression was commensurate with a rise in activity of the transcription factor Sp1, one of the regulators of the APP gene. Furthermore, the increase in APP gene expression in old age was accompanied by an elevation in APP and Aβ proteins. In contrast, APP expression, Sp1 activity, as well as APP and Aβ protein levels were unresponsive to Pb exposure during old age. [144]. The same group analyzed brains of cynomolgus monkeys who had been exposed to Pb as infants, observing elevated levels of APP mRNA, and APP and Aβ protein levels in old monkeys exposed to Pb during brain development [145]. Overall, these data suggested that environmental influences occurring during brain development predetermined the expression and regulation of APP later in life, potentially altering the course of amyloidogenesis [144,145]. The authors observed that lead exposure during brain development of rats and monkeys inhibits DNA-methyltransferases, thus resulting in hypomethylation of the promoters of genes associated with AD, such as *APP*. Whereas AD-related genes were over-expressed late in life, others were repressed, suggesting that early life perturbations result in hypomethylation of some genes as well as hypermethylation of others [144-146]. 

### 3. Studies in Humans

Despite evince of possible epigenetic modifications of AD-related genes obtained in neuronal cell cultures as well as in rodents and primates, epigenetic studies in AD patients are scarce. A recent study performed in lymphocytes (6 AD patients and 6 controls) and post-mortem brain samples (24 AD brains and 10 control brains) of late onset AD patients and matched controls revealed a notably age-specific epigenetic drift associated with unusual methylation patterns in AD samples, supporting a potential role of epigenetic effects in the development of the disease. Particularly, some of the genes that participate in Aβ processing (*PSEN1*, *APOE*) and methylation homeostasis (*MTHFR, DNMT1*) showed a significant interindividual epigenetic variability, which could contribute to AD pathology [147]. 

The promoter of the *APP* gene shows a high GC content (72%), and the frequency of CpG dinucleotides is five times higher than in other eukaryotic promoters, suggesting that its expression might be regulated through methylation of the CpG regions [148]. An initial study of seven potential methylation sites between position -460 and -275 of the *APP* promoter in healthy human brain tissue revealed that none of them was methylated [149]. A subsequent study revealed that the region of the human *APP* promoter upstream of -500 displays complex, tissue-specific patterns of methylation. Furthermore, different patterns of methylation were observed even in DNA from different regions of brain, and these methylation patterns crudely reflected differences in *APP* expression [150]. Tohgi and co-workers identified at least 13 potential methylation sytes in the *APP *promoter region from -226 to -101 in the DNA extracted from post-mortem brain regions of 10 neurologically healthy control subjects. They also observed a reduction with age in the methylcytosine content in this region, suggesting that an age-related demethylation might be linked to A=β deposition in the aged brain [151]. All these studies have been performed in healthy brains and suggest that *APP* expression might be regulated through methylation of its promoter. However, more recent data indicates no difference in methylation of the *APP* gene in AD versus control brains [147].

Recently, Mastroeni and co-workers examined global DNA methylation in monozygotic twins discordant for AD, observing significantly reduced levels of DNA methylation in temporal neocortex neuronal nuclei of the AD twin. These findings are consistent with the hypothesis that epigenetic mechanisms may mediate at the molecular level the effects of life events on AD risk [152]. The same authors analyzed brain tissues from 20 AD patients and 20 cognitively and neurologically normal age-matched controls, observing a markedly decreased nuclear immunoreactivity for 5-methylcytosine in the entorhinal cortex of AD patients, respect to controls. They also observed that nuclear immunoreactivity for the DNA methyltransferase (DNMT1) and for six different components of the MeCP1/MBD2 methylation complex was significantly reduced in the entorhinal cortex of AD subjects than in controls. Overall, these findings indicate epigenetic dysfunctions in AD-vulnerable neurons [153]. 

## PERSPECTIVE

A recent meta-analysis of high quality published studies indicates that plasma Hcy levels are significantly higher in AD patients respect to controls. On the contrary, plasma folate values are significantly reduced in AD patients and the levels of vitamin B12 tend to be lower in AD individuals respect to matched controls [13]. There is also indication from prospective cohort studies that hyper-homocysteinemia and low serum folate values represent risk factors for the development of AD [120-123]. Moreover, studies performed in post-mortem AD and control brains revealed impaired SAM and SAH levels in the first group and suggested a possible inhibition of methyltransferases in the brain of AD individuals  [14,15]. Indeed, a recent study performed on post-mortem AD and control brains revealed a marked reduction of DNA methylation in AD brains, as well as a marked reduction in DNA methyltransferase activities [153]. Overall, there is indication that one-carbon metabolism and DNA methylation are impaired in AD.

Studies performed in mice and in neuronal cell cultures indicate that the depletion of folate and other B vitamins, respectively from the diet or from the media, results in epigenetic modifications of AD-related genes, with a subsequent increased production of presenilin 1, BACE1, and Aβ fragments  [134,136,137,139,140]. Moreover, dietary SAM administration or addiction to the media attenuated the epigenetic changes induced by B vitamin restriction [133, 141]. It was therefore hypothesized that SAM administration could be used as a possible treatment for AD [133]. Recent preclinical and clinical findings demonstrate that dietary supplementation with SAM alleviates a variety of risk factors and hallmarks associated with AD; supporting the notion that nutritional supplementation may represent an important augmentation for therapy in AD [154]. Therefore, it was recommended the need of well-designed intervention trials using measures of dietary supplementation (dietary omega-3 polyunsturated fatty acids and SAM plus B vitamin supplementation) to determine if such supplements will reduce the risk for cognitive decline in very mild AD and mild cognitive impairment [155]. However, there is no yet available data in humans demonstrating that we can use SAM and/or B vitamins to counteract epigenetic modifications of AD-related genes in the brain, and it is my opinion that several considerations must be done in this context.

One of the most important things that we need to clarify is whether or not environmentally induced epigenetic modifications of AD-related genes are reversible and could be modulated through dietary SAM or B vitamin supplementation. The studies performed on rodents and primates exposed to Pb in early life suggest that there is a window of time during brain differentiation when the brain is particularly vulnerable to epigenetic modifications  [144,145]. Particularly, these data suggested that environmental influences occurring during brain development predetermined the expression and regulation of AD-related genes later in life [144,145]. However, no epigenetic modification of AD-related genes was observed when animals were exposed to Pb later in life [144]. These observations should lead to the following reflections: a) What are the vulnerable post-and pre-natal periods in humans when the developing brain is particularly susceptible to epigenetic modifications? b) How many environmental and/or dietary factors are able to induce epigenetic changes in the differentiating brain? c) Are these phenomena reversible? Can a dietary intervention occurring during adulthood restore the methylation pattern of a gene which has been epigenetically modified during brain differentiation? Unfortunately, most of these questions are still unsolved.

The studies performed by the group of Dr. Scarpa suggest that, at least in rodents and in neuronal cell cultures, SAM administration is able to attenuate the epigenetic modification of AD-related genes, particularly *PSEN1*, induced by B vitamin depletion [133,141], leading to the speculation that something similar could happen also in humans. However, what happens in the human brain is still a mystery. There is only 1 published study that analyzed the patterns of methylation of AD-related genes in post-mortem AD and control brains [147]. Even if that study revealed an epigenetic drift in AD subjects, there is no available data in humans that correlates plasma values of folate and Hcy, or brain SAM levels, to the methylation profile of any specific AD gene in the brain. This is another point that requires clarification.

The study by Mastroeni and co-workers revealed a widespread reduction of DNA methylation in post-mortem AD brains, suggesting that epigenetic modifications might contribute to AD pathogenesis [153]. However, the study by Chen and co-workers suggest that, at least in the cell model, the Aβ peptide itself exerts epigenetic properties inducing global DNA hypomethylation and inhibition of DNA methyltransferases [138]. Therefore, is the reduction of DNA methylation observed in post-mortem AD brains [153] a cause of the neurodegenerative process, a consequence of Aβ production and deposition in AD brains, or is it part of a vicious cycle that initially triggers Aβ production and is then perpetuated by Aβ accumulation? We still do not have an answer to this question which is of particular interest during the design of AD treatments based on SAM and B vitamin administration, particularly in the context of when should we treat the patients.

Another important observation comes from the studies by Chan and co-workers [141-143]. These authors observed that in transgenic rodents expressing different variants of the human *APOE *gene, the response to folate depletion and SAM administration, in terms of expression of AD-related genes, was dependent on the *APOE *genotype [141-143]. This is another point to be taken into consideration for the design of AD treatments based on SAM and B vitamin, since we need to know what response must be expected, based on the genotype of the patients. Within this context a recent study performed in an *in vitro* model using colon and breast cancer cells revealed that in colon cancer cells the *MTHFR* 677T mutation was associated with significantly increased genomic DNA methylation when folate supply was adequate or high; however, in the setting of folate insufficiency, this mutation was associated with significantly decreased genomic DNA methylation. In contrast, in breast cancer cells, the *MTHFR* 677T mutation was associated with significantly decreased genomic DNA methylation when folate supply was adequate or high and with no effect when folate supply was low [156]. Similarly, it was shown that the *MTHFR *677C>T polymorphism affects promoter methylation of tumor-specific genes in sporadic colorectal cancer through an interaction with folate and vitamin B12 status. Particularly, high concentrations of serum folate and vitamin B12 levels have been associated with the risk of promoter methylation in tumor-specific genes, and this relationship was modified by the *MTHFR* 677C>T genotypes [157]. A study performed on lymphocyte DNA extracted from 198 healthy subjects revealed that genomic DNA methylation was affected by the *MTHFR *1298 genotypes. Particularly, carriers of the 1298AA wild-type genotype showed lower genomic DNA methylation compared with 1298AC or 1298CC genotypes. Moreover, when DNA methylation was evaluated according to plasma folate status, only 1298AA with low folate levels revealed diminished DNA methylation, and when the two MTHFR polymorphisms were concomitantly evaluated at the low folate status, DNA methylation was reduced only in 1298AA/677TT compared with 1298AA/677CC and 1298CC/677CC genotypes [158]. Overall, there is indication that DNA methylation is a complex trait depending on cell type, B vitamin status, and polymorphisms of genes involved in one-carbon metabolism [156-158]. Unfortunately, there is no available literature concerning the interplay between folate status, polymorphisms of metabolic genes, and the levels of metylation of AD-related genes in the human brain. This is therefore an issue that requires clarification prior to recommend a widespread administration of dietary SAM and folate in dementia and pre-dementia phases. We first need to clarify what subjects, depending on their genotype, would really benefit from such a treatment and what individuals could have no benefits or even adverse consequences.

Concluding, increasing evidence supports interplay between one-carbon metabolism and epigenetic modifications in the brain in the onset of AD (**4**). This is a very promising and exciting field for future investigation as well as for the design of therapeutic and preventive strategies. However, further investigation involving cell cultures, animal models and particularly humans is required for a better comprehension of this complex phenomenon.

## Figures and Tables

**Table 1.** Plasma Folate, Homocysteine (Hcy), Vitamin B12 and Vitamin B6 Levels in AD Patients and Controls

AD Cases/Controls	Total Hcy	Folate	Vitamin B12	Vitamin	Refs
49/52	↑ in AD	No difference	No difference	--	[15]
108/164	↑ in AD	↓ in AD	No difference	--	[16]
17/14	No difference	--	--	--	[17]
19/19	↑in AD	No difference	No difference	--	[18]
74/74	↑in AD	↓ in AD	↓ in AD	--	[19]
277/137	No difference	--	--	--	[20]
71/83	↑ in AD	↓ in AD	No difference	No difference	[21]
27/25	↑ in AD	↓ in AD	AD	[22]
50/57	↑ in AD	↓ in AD	No difference	--	[23]
25/25	↑in AD	--	--	--	[24]
55/74	No difference	No difference	No difference	--	[25]
22/24	↑ in AD	↓ in AD	↓ in AD	No difference	[26]
75/155	↑ in AD	No difference	No difference	--	[27]
11/207	↑ in AD	--	--	--	[28]
21/23	↑ in AD	No difference	No difference	--	[29]
105/102	↑ in AD	--	--	--	[30]
42/50	↑ in AD	--	--	--	[31]
71/44	↑ in AD	--	--	--	[32]
51/40	↑ in AD	↓ in AD	in AD	--	[33]
29/23	↑ in AD	↓ in AD		--	[34]
30/30	--	↑ in AD	--	--	[35]
106/104	↑ in AD	↓ in AD	--	--	[36]

**Table 2.** S-Adenosylmethionine (SAM), S-Adenosylhomocysteine (SAH) and Methionine Adenosyltransferase (MAT) Activity in AD Patients and Controls

AD Cases/Controls	Observation	Refs.
9/13	↓SAM levels in AD CSF	[40]
11/14	↓SAM levels in AD brains ↓ SAH levels in AD brains	[14]
5/5	normal MAT activity in AD brains	[14]
9/10	↓ MAT activity in AD erythrocytes	[41]
30/28	No difference in CSF SAM levels between AD cases and controls No difference in CSF SAH levels between AD cases and controls	[44]
26/29	↑ plasma SAH levels in AD patients ↑ plasma SAM levels in AD patients	[45]
25/25	↓adenosine levels in the plasma of AD patients	[50]
34/43	↑SAH levels in the prefrontal cortex of AD patients ↑SAH IN AD brain inhibits methyltransferases	[51]

**Table 3.** Polymorphisms in Folate/Homocysteine Metabolizing Genes and AD Risk

AD Cases/Controls or (Range)^1^	Gene^2^	Polymorphism	Observation	Refs.
(50-400)	*MTHFR*	677C>T (Ala222Val)	Conflicting results in genetic association studies	[19,30,31,36,56-65]
(50-400)	*MTHFR*	677C>T (Ala222Val)	associated with ↑ plasma Hcy and/or ↓ folate values in AD patients	[19,30,59,61,64]
(50-400)	*MTHFR*	677C>T (Ala222Val)	associated with ↑ AD risk in combination with the *APOE* genotype	[36,61,62,65]
(50-300)	*MTHFR*	1298A>C (Glu429Ala)	Conflicting results in genetic association studies	[31,58,61,66,67]
129/178	*MTHFR*	677C/1298C/1793G (haplotype)	Associated with ↓ AD risk	[71]
223/323	*MTHFR*	-713G>A (promoter region)	Not associated with AD risk	[72]
223/323	*MTHFR*	-393C>A (promoter region)	Not associated with AD risk	[72]
386/375	*RFC1*	80G>A (Arg27His)	Associated with ↑ AD risk	[36]
(150-350)	*MTR*	2756A>G (Asp 919Gly)	Possible association with ↑ AD risk	[99-101]
(70-200)	*TC*	776C>G (Pro259Arg)	Conflicting results in genetic association studies	[61,100,110]
(100-200)	*CBS*	844ins68 (insertion)	Conflicting results in genetic association studies	[30,119]

1 When only 1 reference is quoted the exact number of AD cases/controls is shown. When more than 1 reference is quoted the range of samples in case-control studies is given into brackets (min-max).

2 *CBS*, cystathionine beta-synthase; *MTHFR*, methylenetetrahydrofolate reductase; *MTR*, methionine synthase; *RFC1*, reduced folate carrier; *TC*, transcobalamin.

**Table 4.** Epigenetic Modifications of AD-Related Genes^1^

Experimental Model	Observation	Refs.
Human Neuroblastoma SK-N-SH or SK-N-BE cells	Folate and vitamin B12 deprivation induced epigenetic modifications in the promoter of *PSEN1*, resulting in upregulation of gene expression	[134]
Human neuroblastoma SK-N-BE cells	SAM administration to the media resulted in downregulation of *PSEN1 * expression	[133]
BV-2 mouse microglial cells	SAH administration increased the production of AB peptide likely through induction of hypomethylation of *APP* and *PSEN1 *gene promoters	[137]
Murine cerebral endothelial cells	AB reduces global DNAmethylation whilst increasing DNA methylation of the gene encoding neprilysin	[138]
Rodents	B vitamin deprivation induced hypomethylation in the promoter of *PSEN1*,resulting in upregulation of gene expression	[140]
Rodents and monkeys	Early life exposure to Pb resulted in inhibition of DNA-methyltransferase,hypomethylation of the promoter of *APP* and delayed upregulation of gene expression later in life	[144-146]
Post-mortem human brains	AD brains showed unusual methylation patters, particularly concerning *PSEN1*, *APOE*, *MTHFR*and *DNMT1 * genes	[147]
Post-mortem human brains	AD brains showed a marked reduction of DNA methylation, but no specific gene was analysed in detail	[152,153]

1 *APP*, amyloid precursor protein; *APOE*, apolipoprotein E; *DNMT1*, *DNA* methyltransferase 1; *MTHFR*, methylenetetrahydrofolate reductase; *PSEN1*, presenilin 1.

## ABBREVIATIONS

Aβ: = Amyloid beta
AD: = Alzheimer’s disease
APOE: = Apolipoprotein E
APP: = Amyloid precursor protein
BACE1: = β-secretase
CBS: = Cystathionine β-synthase
CSF: = Cerebrospinal fluid
DNMTs: = DNA methyltransferases
DS: = Down syndrome
GSH: = Glutathione
Hcy: = Homocysteine
MAT: = Methionine adenosyltransferase
5-MTHF: = 5-methyltetrahydrofolate
MTHFR: = Methylenetetrahydrofolate reductase
MTR: = Methionine synthase
MTRR: = Methionine synthase reductase
NFT: = Neurofibrillary tangles
PSEN1: = Presenilin 1
PSEN2: = Presenilin 2
RFC1: = Reduced folate carrier
SAH: = S-adenosylhomocysteine
SAM: = S-adenosylmethionine
SP: = Senile plaques
TC: = Transcobalamin
THF: = Tetrahydrofolate
